# Rac1 GTPase and the Rac1 exchange factor Tiam1 associate with Wnt-responsive promoters to enhance beta-catenin/TCF-dependent transcription in colorectal cancer cells

**DOI:** 10.1186/1476-4598-7-73

**Published:** 2008-09-30

**Authors:** Pinella Buongiorno, Vaijayanti V Pethe, George S Charames, Susmita Esufali, Bharati Bapat

**Affiliations:** 1 Samuel Lunenfeld Research Institute, Mount Sinai Hospital, Toronto, Ontario, M5T 3L9, Canada; 2 Department of Pathology and Laboratory Medicine, Mount Sinai Hospital, Toronto, Ontario, M5G 1X5, Canada; 3 Department of Laboratory Medicine and Pathobiology, University of Toronto, Toronto, Ontario M5S 1A8, Canada

## Abstract

**Background:**

β-catenin is a key mediator of the canonical Wnt pathway as it associates with members of the T-cell factor (TCF) family at Wnt-responsive promoters to drive the transcription of Wnt target genes. Recently, we showed that Rac1 GTPase synergizes with β-catenin to increase the activity of a TCF-responsive reporter. This synergy was dependent on the nuclear presence of Rac1, since inhibition of its nuclear localization effectively abolished the stimulatory effect of Rac1 on TCF-responsive reporter activity. We hypothesised that Rac1 plays a direct role in enhancing the transcription of endogenous Wnt target genes by modulating the β-catenin/TCF transcription factor complex.

**Results:**

We employed chromatin immunoprecipitation studies to demonstrate that Rac1 associates with the β-catenin/TCF complex at Wnt-responsive promoters of target genes. This association served to facilitate transcription, since overexpression of active Rac1 augmented Wnt target gene activation, whereas depletion of endogenous Rac1 by RNA interference abrogated this effect. In addition, the Rac1-specific exchange factor, Tiam1, potentiated the stimulatory effects of Rac1 on the canonical Wnt pathway. Tiam1 promoted the formation of a complex containing Rac1 and β-catenin. Furthermore, endogenous Tiam1 associated with endogenous β-catenin, and this interaction was enhanced in response to Wnt3a stimulation. Intriguingly, Tiam1 was recruited to Wnt-responsive promoters upon Wnt3a stimulation, whereas Rac1 was tethered to TCF binding elements in a Wnt-independent manner.

**Conclusion:**

Taken together, our results suggest that Rac1 and the Rac1-specific activator Tiam1 are components of transcriptionally active β-catenin/TCF complexes at Wnt-responsive promoters, and the presence of Rac1 and Tiam1 within these complexes serves to enhance target gene transcription. Our results demonstrate a novel functional mechanism underlying the cross-talk between Rac1 and the canonical Wnt signalling pathway.

## Introduction

Rac1 GTPase is a member of the Rho family of small GTPases, which play critical roles in the regulation of various cellular processes that include reorganization of the actin cytoskeleton, cell-cycle progression, intercellular adhesion, and gene expression [reviewed in [1]]. Rho family proteins act as molecular switches that cycle between an inactive GDP-bound state and an active GTP-bound state. This cycling is regulated by proteins such as guanine nucleotide exchange factors (GEFs) that activate Rho GTPases by accelerating GDP to GTP exchange, and GTPase activating proteins (GAPs) that deactivate Rho GTPases by increasing their intrinsic rate of GTP hydrolysis. Active, GTP-bound Rho GTPases interact with and activate downstream effector proteins through which they elicit a vast range of cellular effects.

Mounting evidence strongly implicates the dysregulation of Rac1 in cancer development. Elevated expression of Rac1 has been reported in breast, colon, and lung tumours [2]. Rac1 activity is also upregulated in several colon cancer and breast cancer cell lines [3] and is likely attributed to the aberrant expression of upstream Rac1 regulators. Overexpression of the Rac1-specific GEF, T-cell lymphoma invasion and metastasis 1 (Tiam1), has been reported in highly invasive breast tumours [4] and colon carcinomas [5-8], and may contribute to elevated Rac1 signalling in these cancers. One mechanism by which dysregulated Rac1 signalling may promote tumourigenesis is by modulating the activities of various transcription factors, including nuclear factor-kappa B (NF-κB), activator protein-1 (AP-1), and signal transducer and activator of transcription 3 (STAT3), which regulate the transcription of genes involved in tumourigenic events such as cell proliferation, tumour angiogenesis, and cell survival [reviewed in [9]].

Most studies regarding Rho GTPases focus on the cytoplasmic or membrane-associated functions of these proteins. The recent discovery that some Rho GTPases, for example Rac1, possess a nuclear localization signal (NLS) [10] raises the possibility that these GTPases may play important roles in the nucleus as well. In line with this possibility, several binding partners for Rho GTPases were found to have nuclear functions: the Rho effector ROCK2 was reported to increase the histone acetyl transferase (HAT) activity of p300 [11]; the Rac1 and Cdc42 effector Pak1 reportedly phosphorylates histone H3 [12]; and the hematopoietic-specific GEF Vav1 was shown to be a component of transcriptionally active promoter-associated complexes [13]. Despite the emerging significance of Rho GTPases and their interacting partners in the nucleus, their nuclear functions remain poorly understood.

The canonical Wnt signalling pathway coordinates various cellular processes such as cell proliferation and cell fate determination during embryonic development and adult tissue maintenance. Wnt signalling leads to the stabilization and nuclear accumulation of β-catenin, the key mediator of the pathway. In the nucleus, β-catenin associates with members of the T-cell factor/lymphoid-enhancer factor (TCF/LEF) family of HMG-box transcription factors to activate the transcription of several target genes, for example *c-Myc *[14] and *Cyclin D1 *[15,16]. Dysregulation of the Wnt pathway may arise due to mutations in key Wnt pathway components, resulting in the inappropriate formation of a β-catenin/TCF transcription factor complex that constitutively activates Wnt target genes. A considerable amount of evidence has implicated the aberrant formation of this complex as a pivotal event in the development of many cancers, including colorectal cancers[17].

The transcription of Wnt target genes involves a complex network of interactions that converge at TCF-binding elements (TBEs) within Wnt-responsive promoters. In the absence of Wnt signalling, TBE-bound TCFs associate with transcriptional co-repressors, including Groucho/transducin-like enhancer of split (TLE) proteins [18-20] and C-terminal binding protein (CtBP) [21], which maintain chromatin in a transcriptionally inactive state. Upon activation of the Wnt pathway, β-catenin binds to TCF, displacing Groucho/TLE and CtBP co-repressors [21,22], and forming a bipartite transcription factor in which TCF provides the DNA-binding domain and β-catenin provides the transactivation domain. The transactivating function of β-catenin is potentiated by the recruitment of various cofactors, such as Pygopus [23-25], TATA-binding protein (TBP)[26], and cyclic AMP response element-binding protein (CBP)/p300 [27]. Furthermore, functional interactions between components of the Wnt-dependent transcription factor complex and mediators of other signalling pathways have been reported. For example, nuclear components of transforming growth factor-beta (TGF-β)/Smad [28], estrogen receptor [29], and c-jun N-terminal kinase (JNK)/c-jun pathways [30] have been shown to modulate the β-catenin/TCF complex, thereby influencing the temporal and/or tissue-specific pattern of Wnt target gene expression.

Several lines of evidence implicate a role for Rho GTPases, and Rac1 in particular, in the regulation of the Wnt signalling pathway. Both Rac1 and RhoA are activated downstream of the non-canonical Wnt pathway, known as the planar cell polarity (PCP) pathway, and are required for various movements during vertebrate gastrulation [31]. Other studies have reported the interaction between the Rho family GEF Asef and adenomatous polyposis coli (APC), an important component of the Wnt pathway, which leads to increased migration of colorectal cancer cells [32,33]. Recently, we identified Rac1 GTPase as an important regulator of the canonical Wnt signalling pathway in colorectal cancer cells [34]. We showed that Rac1 promotes the accumulation of β-catenin in the nucleus, and synergizes with β-catenin to augment the transcription of Wnt-responsive genes in both Wnt-active colorectal cancer cells and Wnt-inactive HEK293 cells. Mutation of the NLS of Rac1 inhibited its nuclear translocation, and abolished the stimulatory effects of Rac1 on β-catenin/TCF-dependent transcription, indicating that the nuclear presence of Rac1 is critical in mediating canonical Wnt pathway activation.

In this report, we aimed to elucidate the role of Rac1 in the transduction of canonical Wnt signals in colorectal cancer cells. The Rac1-specfic GEF Tiam1 is overexpressed in many colorectal cancers [5,6], and is a transcriptional target of the canonical Wnt signalling pathway [6,35]. We therefore investigated the possible involvement of Tiam1 in Rac1-mediated regulation of the canonical Wnt pathway. Herein, we demonstrate that Rac1 and its activator Tiam1 directly modulate Wnt target gene transcription by associating with the β-catenin/TCF transcription factor complex at Wnt-responsive promoters. Our results demonstrate a novel functional mechanism underlying the interplay between Rac1 and the canonical Wnt signalling pathway, and shed some light on the nuclear roles of Rac1 and Tiam1.

## Results

### Rac1 associates with β-catenin in the nucleus

**Figure 1:**
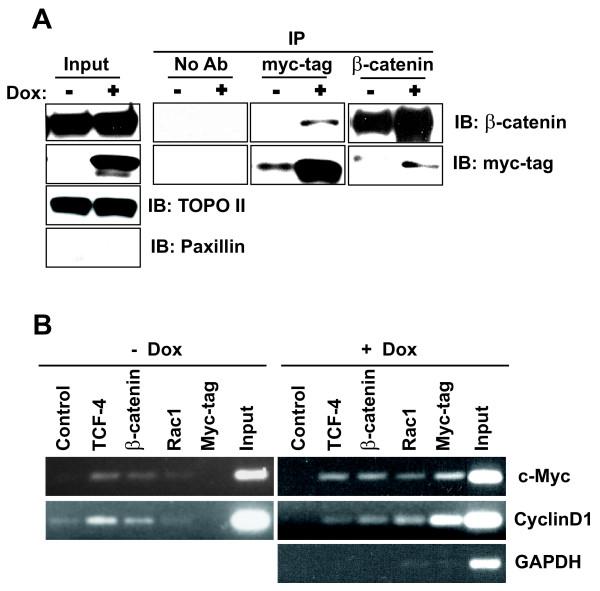
**Active Rac1 associates with Wnt-responsive promoters *in vivo*.** (A) HCT116 cells with stable inducible expression of myc-tagged V12Rac1 were treated with (+) or without (-) 0.1 μg/ml doxycyline (dox) for 24 hours. Nuclear extracts were immunoprecipitated (IP) with myc-tag or β-catenin antibodies and immunocomplexes were analyzed by immunoblotting (IB) with β-catenin and myc-tag antibodies. Nuclear protein (20 μg) was loaded to confirm exogenous expression of myc-tagged V12Rac1 and endogenous β-catenin expression. Topoisomerase II and Paxillin were used as markers for nuclear and cytoplasmic fractions, respectively. (B) HCT116 cells with stable inducible expression of myc-tagged V12Rac1 were treated with (+) or without (-) 0.1 μg/ml doxycycline (dox) for 24 hours. Chromatin was isolated from formaldehyde-fixed cells, and subjected to immunoprecipitation with TCF-4, β-catenin, Rac1, or myc-tag antibodies. IgG or no antibody were used as negative controls. Enriched chromatin was PCR amplified using primers that flank TBEs in *c-Myc *or *Cyclin D1 *promoters. As a negative control, primers specific to the GAPDH promoter, which is devoid of TBEs, were used. Input represents 10% of chromatin used for immunoprecipitation.

We have previously shown that active GTP-bound Rac1, but not the inactive GDP-bound form, synergizes with β-catenin to induce the activity of a TCF-responsive reporter (TOPFlash) [34]. We demonstrated that mutation of the NLS of Rac1 abolished its ability to translocate into the nucleus, as well as its ability to synergize with β-catenin in the activation of the TOPFlash reporter. Furthermore, we previously reported that active Rac1 interacts with β-catenin via co-immunoprecipitation of whole cell lysates. Given the functional interaction between Rac1 and β-catenin, and the dependence of this interaction on the nuclear presence of Rac1, we decided to investigate whether Rac1 and β-catenin might physically associate in the nucleus. To explore this possibility, we generated an HCT116 colorectal cancer cell line with stable and doxycycline-inducible expression of a constitutively active form of Rac1 (V12Rac1). The levels of nuclear β-catenin are inherently high in HCT116 cells due to a β-catenin mutation that results in its stabilization, and as a consequence, constitutive activation of the canonical Wnt pathway. Nuclear extracts were isolated from cells that were treated with or without doxycycline to induce the expression of myc-tagged V12Rac1. Immunoprecipitation with myc-tag antibody co-precipitated β-catenin in doxycycline-treated lysates but not uninduced lysates, even though the expression level of endogenous β-catenin was comparable under both uninduced and induced conditions (Figure 1A). The reverse IP using β-catenin antibody co-precipitated myc-tagged V12Rac1 predominantly in induced nuclear extracts, further confirming the specificity of the interaction. Our results suggest that active Rac1 and β-catenin interact in the nucleus of Wnt-active HCT116 cells, albeit the interaction appears to be weak or transient.

### Rac1 associates with TCF binding elements within Wnt-responsive promoters

The functional interaction between the nuclear pool of Rac1 and β-catenin [34], as well as the physical association between Rac1 and β-catenin in the nucleus, led us to explore the possibility that Rac1 associates with the β-catenin/TCF transcription factor complex at Wnt-responsive promoters. We conducted chromatin immunoprecipitation (ChIP) assays to examine the *in vivo *association of active Rac1 with TCF-binding elements (TBEs) located within the promoters of two well-established Wnt transcriptional targets, *c-Myc *and *Cyclin D1*. For this purpose, we used HCT116 cells with stable inducible expression of V12Rac1 that were treated with or without doxycycline. Immunoprecipitation using TCF-4 or β-catenin antibodies expectedly recovered chromatin that contained TBEs of *c-Myc *or *Cyclin D1 *promoters both in the presence and absence of doxycycline (Figure 1B). Remarkably, under doxycycline-induced conditions, both Rac1 and myc-tag antibodies also enriched for chromatin that contained *c-Myc *or *Cyclin D1 *TBEs. The specificity of these interactions was confirmed when myc-tag antibody did not co-precipitate TBEs in the absence of doxycycline treatment. Consistent with this, in the absence of doxycycline treatment, Rac1-specific antibody enriched for chromatin corresponding to *c-Myc *or *Cyclin D1 *promoters to a lesser extent than with doxycycline treatment. The chromatin recovered by ChIP with Rac1-specific antibody under uninduced conditions was likely co-precipitated with endogenous Rac1. The association between Rac1 and TBEs was specific, since neither Rac1 nor myc-tag antibodies enriched for chromatin containing the promoter region of glyceraldehyde-3-phosphate dehydrogenase (GAPDH), which is devoid of TBEs. Further substantiation of our ChIP results were provided by electrophoretic mobility shift assay (EMSA), which revealed an *in vitro *association between active Rac1 and TBEs [see Additional file 2]. Taken together, our results suggest that Rac1 associates with the β-catenin/TCF-4 transcription factor complex formed at TBE-containing promoters.

### Rac1 enhances the transcription of Wnt target genes

**Figure 2:**
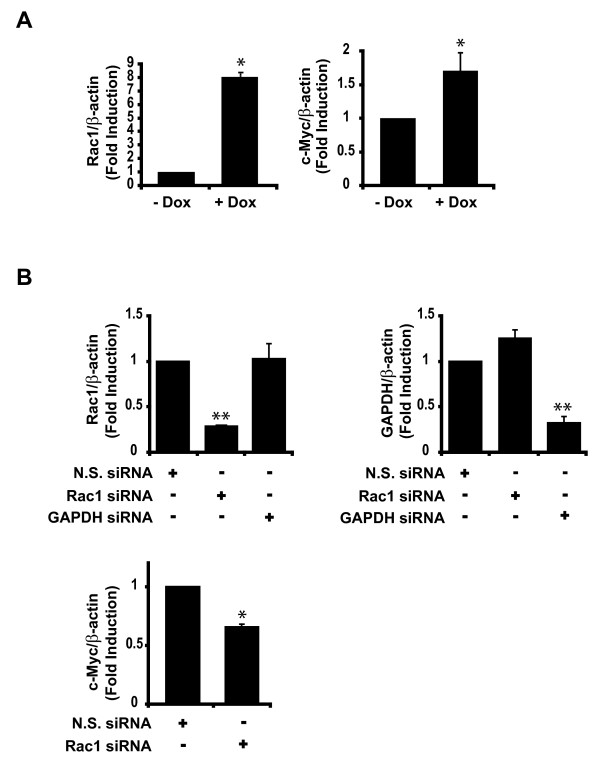
**Rac1 enhances the transcriptional activation of Wnt target genes in HCT116 cells.** (A) HCT116 cells with stable inducible expression of V12Rac1 were serum starved (0.1% FBS) and treated with (+) or without (-) 0.1 μg/ml dox for 24 hours. Total RNA was analyzed by quantitative RT-PCR for the detection of Rac1 and c-Myc mRNA. (B) HCT116 cells were transiently transfected with either non-silencing (N.S.) siRNA, Rac1-specific siRNA, or GAPDH siRNA for 48 hours. Total RNA was analyzed by quantitative RT-PCR for the detection of Rac1 and GAPDH transcript levels (*top*) or c-Myc transcript levels (*bottom*). For quantitative RT-PCR experiments, transcript levels of Rac1, c-Myc, and GAPDH were normalized to transcript levels of β-actin. Bars represent mean ± S.E., *p < 0.05, **p < 0.005.

We have previously shown that transient transfection of V12Rac1 activated the TOPFlash reporter in HCT116 colorectal cancer cells, whereas a dominant negative Rac1 mutant (N17Rac1) inhibited the activity of TOPFlash in these cells [34]. To corroborate our previous observations, we conducted a TOPFlash assay using an HCT116 cell line with doxycycline-inducible expression of V12Rac1 and observed that V12Rac1 expression augmented Wnt signalling in this cell line [see Additional file 3]. We next examined the effect of Rac1 on the regulation of the endogenous Wnt target gene, *c-Myc*. As shown in Figure 2A, doxycycline-induced expression of V12Rac1 in HCT116 cells resulted in ~70% increase in c-Myc transcript levels relative to uninduced cells. To further elucidate the physiological role of Rac1 in the transcription of Wnt target genes, we suppressed the expression of endogenous Rac1 by transfecting HCT116 cells with Rac1-specific siRNA. As shown in Figure 2B (*top left*), ~70% knockdown of Rac1 was achieved using Rac1-specific siRNA, with no suppressive effect on GAPDH transcript levels. The decrease in Rac1 expression was specific since siRNA targeting GAPDH had no effect on the expression of Rac1, but suppressed the expression of GAPDH by ~70% in HCT116 cells (Figure 2B,*top right*). We examined the effects of Rac1 knockdown on the transcript levels of the endogenous Wnt target gene *c-Myc*, and observed a ~40% reduction (Figure 2B, *bottom*). Taken together, our results suggest that Rac1 contributes to the transcriptional activation of endogenous Wnt target genes in colorectal cancer cells. However, we should note that because the effects of Rac1 on the transcript levels of *c-Myc *are modest, other signalling pathways are likely involved in the regulation of endogenous *c-Myc *expression [36-38].

### Tiam1 potentiates Rac1-mediated stimulation of β-catenin/TCF-dependent transcription

**Figure 3:**
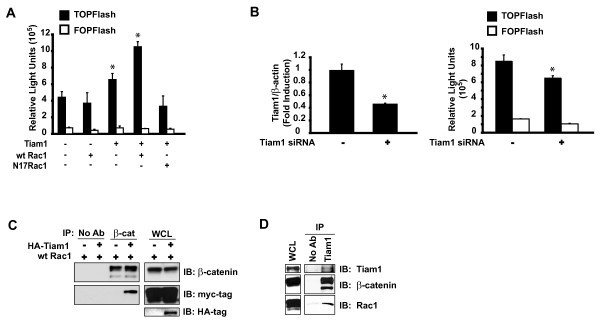
**Tiam1 lies upstream of Rac1-mediated activation of the canonical Wnt pathway.** (A) HCT116 cells were co-transfected with pTOPFlash or pFOPFlash reporter plasmids and pCMVβ-galactosidase, in addition to the indicated expression plasmids: wild-type (wt) Rac1 alone (0.4 μg), Tiam1 alone (0.5 μg), wt Rac1 and Tiam1 in combination (0.4 μg and 0.5 μg, respectively), and N17Rac1 and Tiam1 in combination (0.4 μg and 0.5 μg, respectively). Empty vector was transfected to establish basal TOPFlash activity. Luciferase activity was normalized to β-galactosidase expression, and is expressed as total relative light units (RLU). Results shown are representative for three independent experiments performed in triplicate. Bars represent mean ± S.E. and *p < 0.05. (B) HCT116 cells were transiently co-transfected with either Tiam1-specific siRNA or control, and with either pTOPFlash or pFOPFlash reporter plasmids for 64 hours. Total RNA was analyzed by real time RT-PCR for the detection of Tiam1 transcript levels, which were normalized to transcript levels of β-actin (*left*). Luciferase activity was measured and normalized to β-galactosidase expression, and is expressed as total relative light units (*right*). Bars represent mean ± S.D. and *p < 0.05. (C) HCT116 cells were transfected with indicated plasmids and immunoprecipitated (IP) with β-catenin antibody. Immunoblotting (IB) was performed using β-catenin and myc-tag antibodies to detect myc-tagged wild-type (wt) Rac1. Whole cell lysates (WCL) were immunoblotted with β-catenin, myc-tag, or HA-tag antibodies to confirm expression of β-catenin, wild-type Rac1, or Tiam1, respectively. (D) Whole cell extracts of HCT116 cells were immunoprecipitated (IP) with Tiam1 antibody and analyzed by Western blotting (IB) using Tiam1, β-catenin, or Rac1 antibodies. Endogenous expression of these proteins was confirmed by immunoblotting whole cell lysates with indicated antibodies.

Our previous work has shown that Rac1 must be in its active, GTP-bound state in order to synergize functionally with β-catenin. We demonstrated that only GTP-bound active Rac1, and not the inactive GDP-bound form, could stimulate TOPFlash activity [34]. The requirement for Rac1 activation prompted us to investigate the potential involvement of known regulators of Rac1 activity. Tiam1 is a Rac1-specific GEF [39] that is overexpressed in many colorectal cancers [6,5]. To investigate the possibility that Tiam1 plays a role in Rac1-mediated stimulation of β-catenin/TCF-dependent transcription, we conducted a TOPFlash reporter assay. HCT116 cells transiently transfected with wild-type Rac1 alone had no effect on TOPFlash activity compared to baseline, while Tiam1 alone caused a modest, 1.5-fold increase in TOPFlash activity (Figure 3A). This effect was likely modest due to the inherently high level of Rac1 activation in these cells [34]. When Tiam1 was co-transfected with wild-type Rac1, TOPFlash reporter activity increased by approximately 2.5-fold compared to baseline, similar to the effect of V12Rac1 [see Additional file 3]. In contrast, Tiam1 failed to activate the TOPFlash reporter in the presence of a dominant negative Rac1 mutant that is constitutively inactive (N17Rac1). To further explore the role of Tiam1 in canonical Wnt signalling, we analyzed the effect of Tiam1 knock-down on β-catenin/TCF-dependent transcription. Transfection of HCT116 cells with Tiam1 siRNA caused a reduction in Tiam1 transcript levels by approximately 50% (Figure 3B, *left*), and significantly diminished TOPFlash reporter activity (p < 0.05) compared to control (Figure 3B, *right*). This effect was likely modest due to incomplete knock-down of Tiam1 and due to inherently high levels of Rac1 activation in HCT116 cells, as noted above. Taken together, these results suggest that Tiam1 may act upstream of Rac1-mediated stimulation of the canonical Wnt pathway.

We previously showed that only the active, GTP-bound form of Rac1 associated with β-catenin *in vivo*. Neither ectopic wild-type Rac1, nor the inactive GDP-bound form of Rac1 could physically associate with β-catenin [34]. Given our findings that Tiam1 mediates the stimulatory effects of Rac1 on Wnt target gene transcription, we sought to determine whether Tiam1 could influence the interaction between Rac1 and β-catenin. HCT116 cells were transiently transfected with either myc-tagged wild-type Rac1 alone or myc-tagged wild-type Rac1 together with Tiam1, and cell lysates were immunoprecipitated with β-catenin antibody. In the absence of Tiam1 cotransfection, myc-tagged wild-type Rac1 was not efficiently co-precipitated with β-catenin antibody (Figure 3C). Intriguingly, a strong association between β-catenin and myc-tagged wild-type Rac1 was observed only in the presence of exogenous Tiam1. These results suggest that Tiam1 promotes the association between wild-type Rac1 and β-catenin in HCT116 cells.

### Tiam1 associates with β-catenin in vivo

Since Tiam1 could influence the association between β-catenin and Rac1, we were prompted to determine whether Tiam1 itself could physically interact with β-catenin in colorectal cancer cells. Protein lysates derived from HCT116 cells were immunoprecipitated with Tiam1 antibody to "pull-down" endogenous Tiam1. Interestingly, immunoblotting with β-catenin-specific antibody revealed a robust association between endogenous Tiam1 and endogenous β-catenin (Figure 3D). As expected, Tiam1 antibody co-precipitated Rac1 in these colorectal cancer cells. Our results suggest that endogenous Tiam1 and endogenous β-catenin form a complex in colorectal cancer cells.

### Wnt3a stimulation of 293T cells promotes the convergence of β-catenin and Tiam1/Rac1 pathways

**Figure 4:**
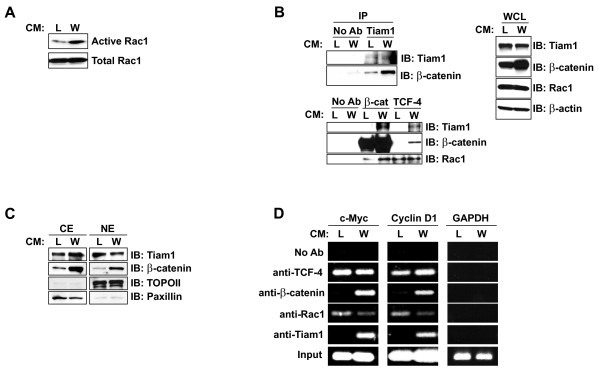
**Wnt3a stimulation of 293T cells promotes the convergence of β-catenin and Tiam1/Rac1 pathways. **293T cells were treated with L-CM (L) or Wnt3a-CM (W) for 18 hours. (A) Activated Rac1-GTP level was determined by GST pull-down assay using the PAK binding domain (PBD), followed by Western blotting analysis using Rac1-specific antibody. Total Rac1 expression was determined by Western blotting analysis of whole cell lysates. (B) Cell extracts were immunoprecipitated (IP) with either Tiam1 antibody (*top*) or β-catenin and TCF-4 antibodies (*bottom*) and analyzed by Western blotting using indicated antibodies. Whole cell lysates (WCL) were analyzed by immunoblotting (IB) to confirm expression of endogenous proteins as shown. Blots were probed with β-actin antibody as a loading control. (C) Cytoplasmic (CE) and nuclear extracts (NE) were analyzed by Western blotting using Tiam1 and β-catenin antibodies. Topoisomerase II and Paxillin antibodies were used to confirm the purity of nuclear and cytoplasmic fractions, respectively. (D) For ChIP assay, chromatin was subjected to immunoprecipitation using TCF-4, β-catenin, Rac1, and Tiam1 antibodies. Enriched chromatin was analyzed by PCR using primers that flank the TBEs in Wnt target genes, *c-Myc *and *Cyclin D1*. GAPDH primers, which flank a region of the GAPDH promoter that is devoid of TBEs, were used as a negative control.

We next wished to determine whether the interaction between Rac1/Tiam1 and the canonical Wnt pathway is Wnt-dependent. To stimulate the canonical Wnt signalling pathway we treated a human embryonic kidney 293T cell line, in which the Wnt signalling pathway is inactive, with Wnt3a-conditioned medium (Wnt3a-CM). Control conditioned medium (L-CM) is devoid of secreted Wnt3a protein, and was used as a negative control. We first confirmed the ability of Wnt3a-CM to stimulate the canonical Wnt pathway in 293T cells by testing for stabilization of endogenous wild-type β-catenin and the induction of TOPFlash activity [see Additional file 4]. As expected, we observed a robust increase in both β-catenin protein expression and TOPFlash activity upon Wnt3a-stimulation of 293T cells. To determine whether Wnt signalling affects the activation of endogenous Rac1, we used a PAK binding domain (PBD) pull-down assay to measure the levels of active GTP-bound Rac1. Treatment of 293T cells with Wnt3a-CM caused an increase in the levels of active GTP-bound Rac1 compared to control (Figure 4A), suggesting that Wnt3a stimulation increases the activation of Rac1.

Next, we wished to determine if Wnt3a stimulation could induce the association of endogenous Tiam1 with endogenous β-catenin and/or TCF-4 in 293T cells. Immunoprecipitation with Tiam1 antibody co-precipitated a considerably higher level of endogenous β-catenin protein in Wnt3a-CM treated cells compared to L-CM treated cells (Figure 4B, *top*). Under reverse conditions, β-catenin antibody co-precipitated endogenous Tiam1 to a much greater extent upon Wnt3a stimulation (Figure 4B, *bottom*). This is not surprising, since β-catenin expression levels increase in response to Wnt3a stimulation, and hence the amount of β-catenin available for binding to Tiam1 is increased as well. Interestingly, we observed an interaction between endogenous TCF-4 and endogenous Tiam1, and this interaction was induced by Wnt3a stimulation (Figure 4B, *bottom*). These results suggest that Wnt3a stimulation promotes the association of Tiam1 with β-catenin and TCF-4. When β-catenin immunoprecipitations were immunoblotted for endogenous Rac1, we found that considerably higher levels of Rac1 were co-precipitated with β-catenin antibody in Wnt3a-CM-treated cells relative to L-CM-treated cells (Figure 4B, *bottom*). Intriguingly, immunoprecipitation with TCF-4 antibody co-precipitated similar levels of endogenous Rac1 in both L-CM- and Wnt3a-CM-treated cells. Therefore, it seems that while Wnt stimulation promotes the interaction between Rac1 and β-catenin, the interaction between TCF-4 and Rac1 is independent of Wnt stimulation.

### Tiam1 is recruited to β-catenin/TCF-4 target gene promoters in a Wnt-dependent manner

Having found that Tiam1 associates with the β-catenin/TCF-4 complex in the presence of a Wnt signal, and that active Rac1 is recruited to Wnt target gene promoters, we aimed to determine whether Tiam1 also associates with Wnt-responsive promoters *in vivo*. Tiam1 is widely characterized as a cytoplasmic protein, and has only recently been found to be localized in the nucleus [40]. We first wished to examine the localization of Tiam1 in 293T cells in the presence and absence of Wnt stimulation. 293T cells were treated with L-CM or Wnt3a-CM for 18 hours and lysates were fractionated into cytoplasmic (CE) and nuclear fractions (NE). We observed expression of endogenous Tiam1 in both cytoplasmic and nuclear fractions of both L-CM- and Wnt3a-CM-treated cells (Figure 4C). As expected, the level of endogenous β-catenin was markedly increased upon Wnt3a-CM treatment in both cytoplasmic and nuclear fractions.

Once the nuclear presence of Tiam1 was established, we asked whether Tiam1 was recruited to Wnt-responsive promoters along with Rac1. We employed ChIP analysis to look for occupancy of TBEs located within the promoters of Wnt target genes *c-Myc *and *CyclinD1 *by Rac1 and Tiam1. For our analyses, we used chromatin derived from 293T cells treated with either L-CM or Wnt3a-CM for 18 hours. As expected, immunoprecipitation of chromatin with TCF-4-specific antibody co-precipitated DNA corresponding to *c-Myc *and *CyclinD1 *TBEs both in the presence and absence of Wnt stimulation (Figure 4D). Also as expected, β-catenin antibody enriched for TBEs only after treatment with Wnt3a-CM. Most notably, endogenous Tiam1 associated with the TBEs of both *c-Myc *and *CyclinD1 *only upon Wnt3a stimulation of cells. Intriguingly, endogenous Rac1 associated with *c-Myc *and *CyclinD1 *TBEs both in L-CM- and Wnt3a-CM-treated cells. None of the ChIPs conducted enriched for chromatin containing the GAPDH promoter, confirming the specificity of these interactions. This data suggests that Tiam1 is recruited to Wnt-responsive promoters in a Wnt-dependent manner, whereas Rac1 is constitutively tethered to TBEs within Wnt target gene promoters.

## Discussion

In this study, we report for the first time that Rac1 GTPase and the Rac1-specific activator Tiam1 are components of the transcriptionally active β-catenin/TCF complex at Wnt-responsive promoters, and that the presence of Rac1 and Tiam1 within this complex serves to enhance Wnt target gene transcription. Furthermore, we provide evidence that Tiam1 mediates the stimulatory effects of Rac1 on the Wnt-responsive transcription factor complex in a Wnt-dependent manner. Taken together, our results offer insights into the mechanisms underlying the cross-talk between Tiam1/Rac1 and the canonical Wnt pathway.

Our findings suggest that Tiam1 and Rac1 play a direct role in enhancing the transcriptional activity of β-catenin on Wnt-responsive promoters. In HCT116 colorectal cancer cells, which harbour constitutive activation of canonical Wnt signalling, ectopically expressed active Rac1 associated with TBEs within the promoters of Wnt-responsive genes *c-Myc *and *Cyclin D1*. Importantly, endogenous wild-type Rac1 was also tethered to these promoters in the Wnt-inactive 293T cell line. While Rac1 was constitutively associated with these promoters, Wnt3a stimulation of cells induced the recruitment of endogenous Tiam1, along with β-catenin. Consistent with our ChIP data, Wnt3a stimulation induced the formation of a complex containing Tiam1 and TCF-4, whereas Rac1 associated with TCF-4 in a Wnt-independent manner. The interaction between β-catenin and Tiam1 was also enhanced upon Wnt3a stimulation of 293T cells. In these cells, the nuclear localization of Tiam1 was observed both in the presence and absence of Wnt3a stimulation, thus it is possible that stimulation of the Wnt pathway forces β-catenin into the nucleus where it forms a complex with nuclear Tiam1. We found that Tiam1 was also localized in the nucleus of HCT116 cells (data not shown). Since high levels of nuclear β-catenin persist in HCT116 cells due to constitutive Wnt pathway activation, it is possible that β-catenin and Tiam1 may associate with each other in a constitutive fashion in the nuclear compartments of these cells. Furthermore, wild-type Rac1 interacted with β-catenin only in the presence of Tiam1, and wild-type Rac1 required the presence of Tiam1 to enhance β-catenin/TCF-dependent transcription. These results suggest that Tiam1 mediates the stimulatory effects of Rac1 on canonical Wnt signalling, and this is supported by the reduction in β-catenin/TCF-dependent transcription in response to Tiam1 knock-down. Since the constitutively active form of Rac1 was capable of interacting with β-catenin [34] and enhanced transcription from Wnt-responsive promoters in the absence of Tiam1 cotransfection, it is possible that the recruitment of Tiam1 to the promoter under physiologic conditions serves to locally activate Rac1 through its GEF activity. In line with this possibility, Wnt3a stimulation of 293T cells promoted the activation of endogenous Rac1.

**Figure 5:**
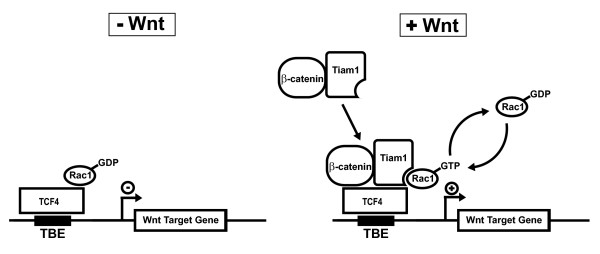
**Proposed model of Rac1/Tiam1-mediated enhancement of β-catenin/TCF-4-dependent transcription.** In the absence of a Wnt signal, Rac1 is tethered to TCF binding elements (TBEs) within Wnt target gene promoters along with TCF-4 and possibly other corepressors in the GDP-bound inactive state. Wnt3a stimulation promotes the formation of β-catenin/Tiam1 complexes that associate with TCF-4 and Rac1 at Wnt target gene promoters. Tiam1 activates Rac1 by mediating GDP to GTP exchange. GTP-bound active Rac1 mediates stimulatory effects on the Wnt-induced transcription factor complex to enhance Wnt target gene transcription.

We propose a model whereby in the absence of a Wnt signal, Rac1 is tethered to Wnt-responsive promoters in an inactive, GDP-bound state along with TCF/LEF and possibly co-repressor proteins (Figure 5). Upon Wnt pathway stimulation, β-catenin forms a complex with Tiam1, which is recruited to Wnt target gene promoters by a promoter-associated complex containing TCF/LEF and inactive, GDP-bound Rac1. We propose that Tiam1 activates Rac1 by catalyzing GDP to GTP exchange, and thereby mediates the stimulatory effects of Rac1 on the Wnt-induced transcription factor complex, resulting in the enhanced transcription of a subset of Wnt target genes, including genes that promote unrestricted cell proliferation such as *c-Myc *and *Cyclin D1*. The contribution of Tiam1-mediated activation of Rac1 to the upregulation of such tumour-promoting genes may represent one mechanism by which the Tiam1/Rac1 signalling module contributes to tumour development in the colon. The status of *Cyclin D1 *as a direct target of the Wnt pathway has recently been disputed by Sansom *et al*., who demonstrated that *Cyclin D1 *was not immediately upregulated following loss of APC in the murine small intestine [41]. More recently, however, β-catenin and other coactivators of Wnt target gene transcription were shown to be recruited to the *Cyclin D1 *promoter in a myoblast cell line in response to Wnt pathway stimulation [42]. This study and several others that provide evidence for the requirement of TBEs for *Cyclin D1 *expression [15,16] suggest that the status of *Cyclin D1 *as a direct Wnt target may be dependent on cell type.

Additional supporting evidence for our model was recently provided by studies comparing tumour formation in Min/+ mice (with a heterozygous nonsense mutation in the *APC *gene that leads to constitutive activation of the canonical Wnt pathway) with different *Tiam1 *backgrounds [5]. Min/+ mice in a Tiam1 ^-^/^- ^background had a significantly reduced susceptibility to develop Wnt-induced intestinal and mammary tumours in comparison to Tiam1 ^+^/^+^/Min/+ mice, and the growth of tumours that did develop was significantly impaired. These studies are consistent with a role for Tiam1/Rac1 in the amplification of nuclear Wnt signalling in colon cancer cells.

Collectively, the results of our study implicate Rac1 and the Rac1-specific GEF Tiam1 as coactivators of β-catenin/TCF-dependent transcription. Consistent with the idea that Rho proteins and their GEFs may act as transcriptional coactivators, a recent study reported a similar role for RhoA, another member of the Rho GTPase family, and the RhoA-specific GEF, Brx. RhoA and Brx were both found to interact with glucocorticoid receptor (GR) at GR-responsive promoters and this interaction increased the transcriptional activity of GR [43]. Furthermore, a recent study demonstrated a direct interaction between Rac1 and activated STAT transcription factors in the nucleus [44], while Tiam1 was shown to interact directly with the transcription factor c-Myc in the nucleus to negatively regulate its transcriptional activity [40]. The finding that traditionally cytoplasmic Rho proteins and their regulators are emerging as important nuclear signal transducers raises the possibility that such proteins may be directly involved in the coordination of cytoplasmic events and changes in gene expression that occur in response to specific upstream signals.

Rac1 enters the nucleus via a recently identified nuclear localization signal (NLS) within its C-terminal polybasic region (PBR) [10]. A previous study has shown that Rac1 promotes the nuclear accumulation of SmgGDS, a GEF for Rho GTPases, through its PBR [45]. It is possible that the PBR of Rac1 similarly facilitates the nuclear accumulation of Tiam1. We speculate that recruitment of Tiam1 to Wnt-responsive promoters may serve to locally catalyze GDP to GTP exchange on promoter-associated Rac1, juxtaposing active, GTP-bound Rac1 and components of the β-catenin/TCF-dependent transcription factor complex. Because GTP-bound Rac1 acts as a molecular switch, it is possible that its presence at the promoter may serve to influence the activation of such components. Previously, active Rac1 and the histone acetyl transferase CBP/p300 were shown to synergize to enhance NF-AT-mediated transcriptional activation in T-cells [46]. It is tempting to speculate that Rac1 might augment β-catenin/TCF-dependent transcription of target genes by influencing the activity of CBP/p300. Alternatively, Rac1 may exert its stimulatory effects on β-catenin/TCF-mediated transcription in colon cancer cells though the downstream effector protein Pak1. A recent study demonstrated that Pak1 modulates the transcription of target genes through direct association with chromatin, and possibly though its ability to modify Histone H3 [12,47]. Intriguingly, a recent study has shown that Pak1 expression is upregulated in adenomas and invasive colon cancers relative to normal colonic epithelium [48]. Whether Pak1 contributes to Tiam1/Rac1-mediated activation of the Wnt pathway warrants further investigation.

*Tiam1 *was recently identified as a Wnt-responsive gene that is upregulated in mouse intestinal tumors and human colon adenomas, and promotes intestinal tumour formation and progression [6,35]. Whether *Tiam1 *is a downstream target of the cross-talk between Wnt and Tiam1/Rac1 signalling pathways is currently being investigated in our lab. Such a finding would suggest the possible existence of a positive feedback loop, whereby aberrant activation of the Wnt pathway induces the transcription of a subset of Wnt target genes such as *Tiam1*, which may mediate the activation of Rac1. As our results suggest, Tiam1-mediated Rac1 activation may feed back to augment nuclear Wnt signalling, leading to sustained Tiam1/Rac1 signalling and as a consequence, the enhanced transcription of target genes that may be important for tumour initiation and/or progression. Future studies will be aimed at exploring the possible existence of such a positive feedback loop.

## Conclusion

The present study sheds some light on the mechanism underlying the cross-talk between the Tiam1/Rac1 signalling pathway and the canonical Wnt pathway in colorectal cancer cells. Our results also suggest a novel nuclear role for Rac1 and its activator Tiam1. We have demonstrated for the first time that Tiam1/Rac1 associate with the β-catenin/TCF transcription factor complex at Wnt-responsive promoters to enhance Wnt target gene transcription. Future studies aimed at elucidating the mechanisms that underlie the ability of Tiam1/Rac1 to directly regulate β-catenin/TCF-dependent gene transcription, as well as the identification of novel chromatin targets of Tiam1/Rac1, will provide important insights into the novel roles of these proteins in the nucleus.

## Methods

### Cell culture

HCT116 cells (ATCC) were cultured in McCoy's 5A media supplemented with 10% fetal bovine serum (FBS). HCT116 cells with stable inducible expression of V12Rac1 or N17Rac1 were cultured in McCoy's 5A media supplemented with 10% FBS, 5 μg/ml blasticidin and 300 μg/ml zeocin. 293T cells were cultured in Dulbecco's modified Eagle's medium (DMEM) supplemented with 10% FBS. L-cells and Wnt3a-expressing L-cells (gifts from Dr. Liliana Attissano, University of Toronto) were maintained in DMEM supplemented with 10% FBS. Wnt3a-expressing L-cells were additionally supplemented with 400 μg/ml G418. For the preparation of L- and Wnt3a-CM, CM was collected from cultured parental L-cells or Wnt3a-expressing L-cells and was diluted to 50% in serum-free DMEM. All cell lines were cultured at 37°C in a humidified atmosphere of 5% CO_2_.

### Antibodies

Antibodies used for co-immunoprecipitation analyses were as follows:**β **-catenin (H-102), myc-tag (A-14), Tiam1 (C-16), TCF-4 (H-125) (all from Santa Cruz Biotechnology, Santa Cruz, CA). Antibodies used for immunoblotting were as follows: β-catenin (Transduction Laboratories, BD Biosciences), myc-tag (9E10, Santa Cruz Biotechnology), Rac1 (Transduction Laboratories, BD Biosciences), Topoisomerase II (Oncogene Research Products, Boston, MA), Paxillin (Transduction Laboratories, BD Biosciences), β-actin (Sigma-Aldrich, St. Louis, MO) and HA-tag (HA-7, Sigma-Aldrich). Antibodies used for chromatin immunoprecipitation assays were as follows: TCF-4 (H-125, Santa Cruz Biotechnology) or TCF-4 (6H5-3, Upstate Biotechnology, Lake Placid, NY), β-catenin (H-102, Santa Cruz Biotechnology), Rac1 (C-14, Santa Cruz Biotechnology), myc-tag (A-14, Santa Cruz Biotechnology), and Tiam1 (C-16, Santa Cruz Biotechnology).

### Plasmids

2 × myc-tagged V12Rac1/pcDNA3.1 was purchased from UMR cDNA Resource Center (Rolla, MO). pcDNA6/TR encoding Tet repressor and pcDNA4/TO inducible expression vector were included in the T-REx system, which was purchased from Invitrogen Life Technologies. To generate the doxycycline inducible 2 × myc-tagged V12Rac1/pcDNA4/TO construct, 2 × myc- V12Rac1/pcDNA3.1 was digested with Hind III and Xba I and the fragment encoding 2 × myc-V12Rac1 was subcloned into Hind III and Xba I-digested pcDNA4/TO. pTOPFlash and pFOPFlash luciferase constructs, and dominant-negative TCF-4 construct were gifts from Dr. Benjamin Alman (Hospital for Sick Children, Toronto). The plasmid encoding myc-tagged wild-type Rac1 was a gift from Dr. Tony Pawson (SLRI, Mount Sinai Hospital, Toronto). Wild-type Tiam1 construct was a gift from Dr. John Collard (Netherlands Cancer Institute, Netherlands).

### Generation of stable inducible cell lines

T-REx system (Invitrogen Life Technologies) was employed to generate HCT116 cell lines with stable inducible expression of V12Rac1 according to manufacturer's instructions. HCT116 cells were transfected in 10-cm dishes with 13 μg pcDNA6/TR per plate using LipofectAMINE 2000 (Invitrogen) transfection reagent. Forty-eight hours after transfection, cells were re-seeded into 15-cm plates at low density and transfectants were selected for in the presence of 5 μg/ml blasticidin. Resistant colonies were screened by Western blotting. Positive clones were subsequently transfected with 2 × myc-V12Rac1/pcDNA4/TO in 10-cm dishes as described above and selected for with 300 μg/ml zeocin. To screen resistant colonies, cells were treated with either 1 μg/ml doxycycline (dox) or vehicle (water), and protein lysates were harvested after 24 hours and analyzed by Western blotting. Positive clones were maintained in blasticidin and zeocin.

### Coimmunoprecipitations, cell fractionation, and Western blotting

For analysis of nuclear lysates by coimmunoprecipitation, nuclear extracts were isolated using Nuclear Complex Co-IP Kit (Active Motif, Carlsbad, CA, USA) and immunoprecipitations were carried out according to manufacturer's instructions. Nuclear extracts (500 μg) were subjected to immunoprecipitation using 3 μg antibody. Coimmunoprecipitation analysis of HCT116 whole cell lysates was performed as described [34]. Whole cell lysates (1000 μg) were subjected to immunoprecipitation using 3 μg antibody. For analysis of 293T cells by coimmunoprecipitation, whole cell lysates (500 μg) were immunoprecipitated with 3 μg antibody. Immunocomplexes were resolved on 10% SDS-PAGE gel, and analyzed by Western blotting using indicated antibodies. For cell fractionation analysis, the NE-PER kit (Pierce, Rockford, IL) was used according to manufacturer's instructions.

### Chromatin immunoprecipitation (ChIP) assay

ChIP assay was performed using a ChIP-IT kit (Active Motif, Carlsbad, CA, USA) according to manufacturer's instructions. Sonication conditions were 15 × 20s to 20 × 20s, and generated 200–1000 bp fragments. Chromatin was immunoprecipitated using 4 μg indicated antibodies. Recovered DNA was analyzed by PCR using primer pairs that flank TBEs in *c-Myc *or *Cyclin D1 *promoters. GAPDH primers, which flank a region of the GAPDH promoter that is devoid of TBEs, were used as a negative control. The *c-Myc *primers were: forward, 5'-GCTCTCCACTTGCCCCTTTTA-3', and reverse, 5'-GTTCCCAATTTCTCAGCC-3'. The *Cyclin D1 *primers were: forward, 5'-GACTACAGGGGAGTTTTGTTG-3', and reverse, 5'-TCGGCTCTCGCTTCTGCTG-3'. The *GAPDH *primers were: forward, 5'-TACTAGCGGTTTTACGGGCG-3', and reverse, 5'-TCGAACAGGAGGAGCAGAGA-GCGA-3'. PCR products were run on a 2% agarose gel and visualized with ethidium bromide. Additional data describing Electrophoretic Mobility Shift Assay (EMSA) are described in [Additional file 1].

### Reporter gene assay

Cells were transiently transfected using LipofectAMINE 2000 reagent (Invitrogen Life Technologies) according to manufacturer's instructions. Cells were seeded in 24-well plates at a density of 2 × 10^5 ^cells per well. Twenty-four hours post-seeding, cells were transfected with 0.1 μg luciferase reporter gene plasmid containing either four wild-type (pTOPFlash) or mutant (pFOPFlash) TCF binding elements repeated in tandem upstream of a luciferase reporter gene. Cells were co-transfected with various amounts of plasmid DNA, as indicated in figure legends. The total amount of DNA per transfection was held constant at 1.03 μg total plasmid DNA by co-transfection with appropriate amounts of empty vector (pcDNA3.1). Cells were co-transfected with 0.03 μg of plasmid encoding β-galactosidase (pCMVβ-gal) as an internal control. Luciferase and β-galactosidase activities were measured 24 hours after transfection. Luciferase values were normalized to β-galactosidase expression. For statistical analyses, unpaired Student's *t*-test was performed. Additional reporter gene assay methods are described in [Additional file 1].

### Quantitative Reverse Transcriptase (RT)-PCR

Total RNA was isolated using an RNeasy Mini kit (Qiagen, Valencia, CA) and analyzed by real-time RT-PCR as described [49]. For RNAi experiments, cells were transfected with either: SMARTpool Rac1-specific siRNA, ON-TARGETplus SMARTpool Tiam1-specific siRNA, siCONTROL non-silencing siRNA as a control, GAPDH-specific siRNA as a control (Dharmacon, Lafayette, CO.) or mock transfected using LipofectAMINE 2000 according to manufacturer's protocol. After 48 hours (Rac1 siRNA experiments) or 64 hours (Tiam1 siRNA experiments), total RNA was isolated and analyzed by real-time RT-PCR. The following primer pairs were used to amplify cDNA: c-Myc forward, 5'-GCCAAGCTCGTCTCAGAGAAG-3', and reverse, 5'-CAGAAGGTGATCCAGACTCTG-3'; Rac1 forward, 5'-ATGCAGGCCATCAAGTGTGTG-3', and reverse, 5'-TTACAACAGCAGGCATTTTCTCT-3'; Tiam1 forward, 5'-AGACGTACTCAGGCCATGTC-3', and reverse, 5'ACCCAAATGTCGCAGTCAGG-3'; β-actin forward, 5'-ATCATGTTTGAGACCTTCAA-3', and reverse, 5'-CATCTCTTGCTCGAAGTCCA-3', and; GAPDH forward, 5'**-**ACCACAGTCCATGCCATCAC-3', and reverse, 5'-TCCACCACCCTGTTGCTGTA-3'. For statistical analyses, unpaired Student's *t*-test was performed.

### Rac1 activation assay

Endogenous active GTP-bound Rac1 levels were determined using a PAK binding domain (PBD) pull-down assay (EZ-Detect Rac1 activation kit, Pierce Biotechnology, Rockford, IL) according to the manufacturer's protocol. Briefly, cells were washed in cold PBS and lysed on ice in 500 μl lysis buffer. Total lysates were cleared by centrifugation at 16000 × g for 15 mins. 100 μL of the lysate was kept for protein quantitation of total Rac1 protein. The remaining lysate was incubated for 1 hour at 4°C with 20 μg PBD agarose beads. Precipitated complexes were washed three times with excess lysis buffer. After the final wash, the supernatant was discarded and the beads were suspended in 40 μl of 2 × Laemmli sample buffer. Levels of total Rac1 and active Rac1 were then analyzed by Western blotting.

## Competing interests

The authors declare that they have no competing interests.

## Authors' contributions

PB performed most of the experiments, contributed to the design of the study and analysis of data, and drafted the manuscript. VVP contributed to the luciferase reporter assays, contributed to the generation of stable inducible cell lines, and performed EMSA and Rac1 activation assays. GSC performed Tiam1 siRNA experiments. SE contributed to the design of the study and the analysis of data. BB contributed to the design of the study, analysis of data, and editing of the manuscript. All authors read and approved the final manuscript.

## Supplementary Material

Additional file 1 Supplementary Information. This contains the Supplementary Methods information and the Supplementary figure legends. Click here for file

Additional file 2 Supplementary figure 1. Active Rac1 binds to the concensus TBE *in vitro *as shown by electrophoretic mobility shift assay (EMSA). Click here for file

Additional file 3 Supplementary figure 2. Active Rac1 enhances transcription from Wnt-responsive promoters. Click here for file

Additional file 4 Supplementary figure 3. Wnt3a-CM stimulates the canonical Wnt pathway in 293T cells. Click here for file
