# Supplementary material for: Rac1 GTPase and the Rac1 exchange factor Tiam1 associate with Wnt-responsive promoters to enhance beta-catenin/TCF-dependent transcription in colorectal cancer cells
Source: Mol Cancer. 2008 Sep 30;7:73. doi: 10.1186/1476-4598-7-73 (PMC2565678; doi:10.1186/1476-4598-7-73)
Supplement: Additional file 1 — Supplementary Information. This contains the Supplementary Methods information and the Supplementary figure legends. [file 1476-4598-7-73-S1.doc]

**Supplementary information:**

**Supplementary methods**

***Electrophoretic Mobility Shift Assay***

Single-stranded oligonucleotides corresponding to the consensus TBE were labelled using Biotin 3’-end labeling kit (Pierce, Philadelphia, PA). Biotin-end labelled single-stranded oligonucleotide probes were annealed by heating equimolar amounts of complementary strands to 95^0^C for 5 mins and slowly cooling the reaction mixture to room temperature. Whole cell lysates derived from HCT116 cells with stable inducible expression of V12 Rac1 were prepared using RIPA lysis buffer (0.5% Nonidet P-40, sodium deoxycholate, 0.1% SDS, 1×PBS, Complete Mini EDTA-free protease inhibitor tablet (Roche Applied Bioscience)). For binding reaction and detection, we used LightShift Chemiluminescent EMSA and Chemiluminiscent Nucleic Acid Detection module respectively (Pierce). Briefly, 20 fmol of labelled probe was incubated with extract (~3 μg protein) in a total volume of 20 μl for 20 mins at room temperature with 1×binding buffer (10mM Tris, 50 mM KCl, 1mM DTT; pH 7.5. Final concentrations of binding reaction components, namely, glycerol, MgCl2, and NP-40 were adjusted according to manufacturer’s recommendations). To prevent non-specific binding of nuclear proteins, 100 ng of poly dI:dC was added and the specificity of retarded bands was confirmed by including 100× excess of unlabelled oligonucleotides. For EMSA interference experiments, 2 μg of mouse monoclonal antibodies specific to β- catenin (Transduction Labs, BD Biosciences), or Rac1 (Transduction Labs, BD Biosciences) were used. Protein-DNA complexes were separated from unbound DNA using 6% (w/v) native PAGE and run in 0.5X TBE. Sequences of probes were as follows: wild-type TBE forward, 5’-ACATACTTTCAAAGTTCTGTA-3’, and reverse, 5’-TACAGAACTTTGAAAGTATGT-3’; mutant TBE forward, 5’-ACATACTGCCAAAGTTCTGTA-3’, and reverse, 5’-TACAGAACTTTGGCAGTATGT-3’.

***Reporter gene assay***

Reporter gene assays were performed as described [1]. For assays using stable inducible cell lines, cells were transfected with luciferase reporter and pCMV-β-galactosidase constructs for 24 h, then treated with 0.05 μg/ml to 1.0 μg/ml doxycycline or vehicle (water). Luciferase and β-galactosidase activities were measured 24 hours after doxycycline treatment. All luciferase values were normalized to β-galactosidase expression. For statistical analyses, unpaired Student’s *t*-test was used.

**Supplementary figure legends**

**Figure 1.** Active Rac1 binds to the concensus TBE *in vitro* as shown by electrophoretic mobility shift assay (EMSA). A biotin-labelled double-stranded oligonucleotide probe corresponding to the consensus TBE was incubated with whole cell extracts derived from doxycycline-treated HCT116 cells with stable inducible expression of V12Rac1. The consensus TBE showed a strong shift upon incubation with the extract, and this shift was markedly diminished by co-incubating with unlabelled TBE probe, but not with unlabelled probe corresponding to the mutated TBE. The retarded band thus presumably corresponds to a complex of proteins containing TCF-4. The binding reaction was also incubated with antibodies specific to β-catenin, Rac1, or IgG (negative control) prior to separation on 8% native PAGE. β-catenin but not IgG antibody interfered with the formation of the complex, which is consistent with previous studies in which binding of β-catenin antibody inhibited the β-catenin/TCF-4 interaction in interference assays [2, 3]. Incubation with Rac1 antibody also interfered with complex formation, suggesting that Rac1 is a component of the complex as well.

**Figure 2.** Active Rac1 enhances transcription from Wnt-responsive promoters. HCT116 cells with stable inducible expression of V12Rac1 were co-transfected with pTOPFlash (black bars) or pFOPFlash (white bars) reporter plasmids and an internal control (pCMVβ-gal) either alone or in combination with dominant negative TCF-4 plasmid (DNTCF4). Cells were induced 24 hours post-transfection with 0.05 μg/ml (+), 0.1 μg/ml (++), or 1 μg/ml (+++) of doxycycline and assayed for luciferase activity after 24 hours. Luciferase activity was normalized to β-galactosidase expression, and is expressed as total relative light units (RLU). Results shown are representative for three independent experiments performed in triplicate. Bars represent mean ± S.E. *P < 0.005, significantly different from Dox (-) control group. **P < 0.005, significantly different from Dox (+) group.

**Figure 3.** Wnt3a-CM stimulates the canonical Wnt pathway in 293T cells. (A) Treatement of 293T cells with Wnt3a-CM leads to stabilization of β-catenin protein. 293T cells were treated with L-CM (L) or Wnt3a-CM (W) for 2, 6, 10, or 18 hours. Whole cell lysates were analyzed by immunoblotting using antibodies that recognize endogenous β-catenin or endogenous β-actin to indicate equal protein loading. (B) Wnt3a-CM stimulation of 293T cells causes an increase in TOPFlash reporter activity. 293T cells were co-transfected with pTOPFlash (white bars) or pFOPFlash (black bars) reporter plasmids and an internal control (pCMVβ-galactosidase). After 24 hours, cells were treated with either L-CM (L) or Wnt3a-CM (W) and were assayed for luciferase activity after 18 hours. Luciferase activity is expressed as total relative light units (RLU) and bars represent the mean ± S.E. of luciferase activity normalized to β-galactosidase expression and *p < 0.0005.

**References**

1. Esufali S, Bapat B: **Cross-talk between Rac1 GTPase and dysregulated Wnt signaling pathway leads to cellular redistribution of beta-catenin and TCF/LEF-mediated transcriptional activation.** *Oncogene* 2004, **23:**8260-8271.

2. Li J, Mizukami Y, Zhang X, Jo WS, Chung DC: **Oncogenic K-ras stimulates Wnt signaling in colon cancer through inhibition of GSK-3beta.** *Gastroenterology* 2005, **128:**1907-1918.

3. Rahmani M, Read JT, Carthy JM, McDonald PC, Wong BW, Esfandiarei M, Si X, Luo Z, Luo H, Rennie PS, McManus BM: **Regulation of the versican promoter by the beta-catenin-T-cell factor complex in vascular smooth muscle cells.** *J Biol Chem* 2005, **280:**13019-13028.
