# Supplementary material for: Rac1 GTPase and the Rac1 exchange factor Tiam1 associate with Wnt-responsive promoters to enhance beta-catenin/TCF-dependent transcription in colorectal cancer cells
Source: Mol Cancer. 2008 Sep 30;7:73. doi: 10.1186/1476-4598-7-73 (PMC2565678; doi:10.1186/1476-4598-7-73)
Supplement: Additional file 2 — Supplementary figure 1. Active Rac1 binds to the concensus TBE in vitro as shown by electrophoretic mobility shift assay (EMSA). [file 1476-4598-7-73-S2.pdf]

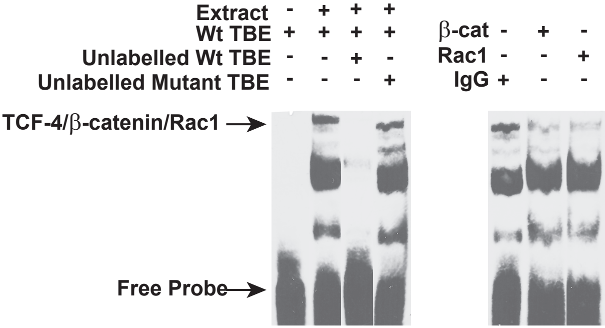

Extract - + + +
Wt TBE + + + +
Unlabelled Wt TBE - - + -
Unlabelled Mutant TBE - - - +
β-cat - + -
Rac1 - - +
IgG + - -
TCF-4/β-catenin/Rac1
Free Probe
