# Supplementary figures and images for: Rac1 GTPase and the Rac1 exchange factor Tiam1 associate with Wnt-responsive promoters to enhance beta-catenin/TCF-dependent transcription in colorectal cancer cells
Source: Mol Cancer. 2008 Sep 30;7:73. doi: 10.1186/1476-4598-7-73 (PMC2565678; doi:10.1186/1476-4598-7-73)

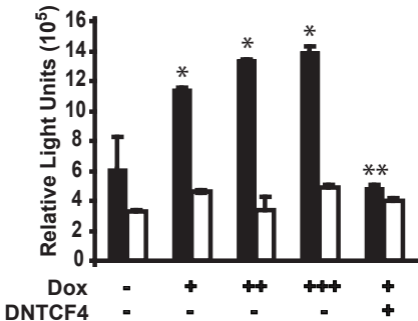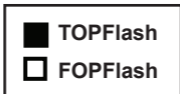

Supplement: Additional file 3 — Supplementary figure 2. Active Rac1 enhances transcription from Wnt-responsive promoters. [file 1476-4598-7-73-S3.pdf]

**A**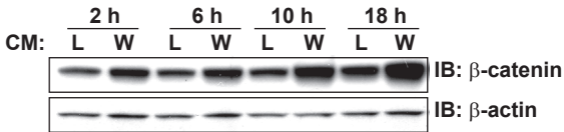**B**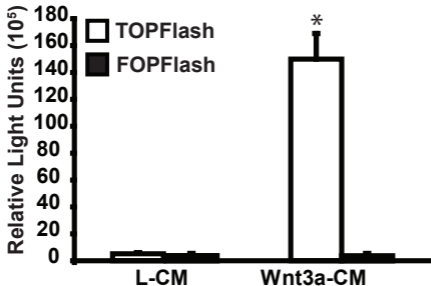

Supplement: Additional file 4 — Supplementary figure 3. Wnt3a-CM stimulates the canonical Wnt pathway in 293T cells. [file 1476-4598-7-73-S4.pdf]
